# Global spatial errors and local feature errors drive oculomotor learning

**DOI:** 10.1167/jov.26.8.3

**Published:** 2026-08-03

**Authors:** Frauke Heins, Markus Lappe

**Affiliations:** 1 Institute for Psychology and Otto-Creutzfeldt Center for Cognitive and Behavioral Neuroscience, University of Muenster, Muenster, Germany

**Keywords:** saccade adaptation

## Abstract

Systematic intra-saccadic displacements of the saccade target object induce oculomotor learning. This type of manipulation remains undetected because of saccadic suppression of displacement. The subtle intra-saccadic manipulation causes the target object to appear at a retinal position that is inconsistent with the computed displacement of visual space during the saccade, leading to the computation of a postdicted motor error and corresponding adaptive changes to the saccade amplitude and the perceived location of objects in space. Adaptive changes to the saccade amplitude or direction have also been observed following obvious intra-saccadic manipulations, but very little is known about the characteristics of this learning process or its underlying mechanisms. The current study therefore compares oculomotor learning in response to subtle and obvious changes of the stimulus display. By measuring their effect on object localization and saccade gain, we were able to show that two different kinds of sensory error can induce oculomotor learning: a global spatial error and a local feature error. While experiencing the former leads to the typical adaptation-induced shift in perceived object location, experiencing the latter does not. On top of that, we demonstrate that adaptive adjustments to the oculomotor behavior do not necessarily rely on those sensory errors. Instead, the overt oculomotor behavior minimizes a task error.

## Introduction

Saccades move our retina with high speed across the visual scene and bring a target object from the visual periphery into foveal vision. Because these ballistic movements are so fast, visual information obtained during the eye movement cannot be used to apply changes to the trajectory of the current saccade. Instead, saccade accuracy has to be evaluated after eye movement, and potential errors are used to adjust future saccades. It is hypothesized that this post-saccadic evaluation of saccade accuracy requires that the object near the fovea first has to be recognized as the target object that has been selected before movement onset (Collins, Vergilino-Perez, Beauvillain, & Doré-Mazars, 2007; Madelain, Harwood, Herman, & Wallman, 2010; Schütz & Souto, 2015), and that this process considers position as well as surface feature information about the objects in the scene (Richard, Luck, & Hollingworth, 2008). The pre-saccadic target object is assumed to be defined by a specific combination of features, for example, its position, shape, or color. This feature combination might be stored in visuospatial working memory, and, after the saccade, be compared with the feature information extracted from the object near the fovea. If its features and the features stored in the visuospatial working memory match, the post-saccadic target recognition is supposed to be successful, and trans-saccadic object correspondence might be established (Currie, McConkie, Carlson-Radvansky, & Irwin, 2000; McConkie & Currie, 1996; Tas, Moore, & Hollingworth, 2012; Van Der Stigchel & Hollingworth, 2018).

Post-saccadic target recognition enables the evaluation of saccade accuracy. It is a prerequisite for detecting a post-saccadic error and is therefore necessary to determine whether any adaptive modifications should be made to future saccades. Adaptive changes are required if the saccade fails to align the fovea with the target object, as is the case, for example, in nerve palsy (Abel, Schmidt, Dell’Osso, & Daroff, 1978; Kommerell, Olivier, & Theopold, 1976). However, such a post-saccadic error can also be induced artificially. This can be done by repeatedly manipulating the position of the target object during the saccade. The target object can be shifted in or against saccade direction. The saccade then under- or overshoots the target object, respectively (McLaughlin, 1967). The target shift remains undetected because of saccadic suppression of displacement (Bridgeman, Hendry, & Stark, 1975; Li & Matin, 1990). This phenomenon might be supported by a strong prior assumption of a stable external world during saccadic eye movements (Atsma, Maij, Koppen, Irwin, & Medendorp, 2016; Choi, Chiu, Ferreira, & Golomb, 2025; Niemeier, Crawford, & Tweed, 2003), which is only abandoned if the discrepancies between the pre- and post-saccadic images are too large (Atsma et al., 2016; Choi et al., 2025). Despite participants being unaware of the intra-saccadic manipulation, adaptive changes to the saccade amplitude develop and gradually decrease the post-saccadic distance between the fovea and the target object (Deubel, Wolf, & Hauske, 1986). This learning process is termed saccadic adaptation. Its time course is often modeled with an exponential function because, after a rapid initial change to the amplitude, the process continues to develop more slowly until a new steady state is reached (Deubel et al., 1986; Scudder, Batourina, & Tunder, 1998; Straube, Fuchs, Usher, & Robinson, 1997). Even after this new steady state has been reached, saccadic adaptation does not compensate for the full extent of the intra-saccadic target displacement (Semmlow, Gauthier, & Vercher, 1989; Straube et al., 1997; Miller, Anstis, & Templeton, 1981). Another important hallmark of saccadic adaptation is its effect on the perceived position of objects. Before the repeated exposure to intra-saccadic target displacements, the spatial position of a localization stimulus presented during the saccade preparation period is reported accurately. However, after exposure to the double-step paradigm, the perceived location of objects shifts in the direction of the target displacement, ultimately leading to a localization error (Awater, Burr, Lappe, Morrone, & Goldberg, 2005; Bahcall & Kowler, 1999; Bruno & Morrone, 2007; Collins, Doré-Mazars, & Lappe, 2007; Heins & Lappe, 2022a; Schnier, Zimmermann, & Lappe, 2010; Zimmermann & Lappe, 2009). This effect pertains both to objects that are presented during fixation and during saccade preparation, but is more pronounced for the latter (Collins et al., 2007; Zimmermann & Lappe, 2010; Schnier et al., 2010).

The nature of the error signal underlying saccadic adaptation has been debated. Initially, the visual error was proposed. It describes the post-saccadic distance between the target object and the fovea. The visual error was rejected because it is not consistent with the natural saccade hypometria (Becker, 1989; Henson, 1979) and the incomplete adjustment of the saccade amplitude to the intra-saccadic target displacement (Deubel et al., 1986; Noto & Robinson, 2001; Straube et al., 1997; Wallman & Fuchs, 1998). Thus, another error signal was proposed: the prediction error. According to this approach, an error is identified if the predicted and actual retinal eccentricity of the target object do not match (Bahcall & Kowler, 1999; Bahcall & Kowler, 2000; Collins & Wallman, 2012; Wong & Shelhamer, 2011). The prediction is supposed to rely on an estimate of the pending saccade, derived from the efference copy (Cavanaugh, Berman, Joiner, & Wurtz, 2016; Crapse & Sommer, 2008; Melcher & Colby, 2008; Wurtz, 2018). However, the prediction error presents with several weaknesses. 1) Once the learning process is complete and a steady state has been reached, the prediction error should be nullified. During this state, the predicted post-saccadic target position and the actual position of the displaced target should match. However, Masselink and Lappe (2021) demonstrated that this is not the case. 2) If the estimated and actual post-saccadic retinal target eccentricity are the same, learning has optimized the prediction. However, because the magnitude of this post-saccadic retinal target eccentricity is irrelevant, optimized predictions do not necessarily correspond with optimized saccade accuracy. 3) The prediction error could indicate the distance by which the world moved intra-saccadically, which would be inconsistent with saccadic suppression of displacement. Thus, Masselink and Lappe (2021) proposed the postdiction error, which overcomes the limitation of the prediction error. According to this new model, the post-saccadic target position and the computed displacement of visual space are combined to postdict the post-saccadic target position into pre-saccadic coordinates after each eye movement. The motor error is then evaluated retrospectively as the difference between the postdicted target position and the motor command. Because the postdicted motor error operates under the assumption of a stable intra-saccadic world, the cause of the post-saccadic error is either attributed to an inaccurate localization of the pre-saccadic target or to erroneous motor performance. Accordingly, the motor error is nullified via the adaptation of three synaptic gains that affect different sensorimotor transformations: 1) the visual gain, which transforms the pre-saccadic retinal input into the target position, 2) the motor gain, which describes the transformation of the spatial target position into a motor command, and 3) the corollary discharge (CD) gain, which describes the transformation of the efference copy of the motor command into the computed displacement of visual space. The plasticity of these different gains is hypothesized to cause the gradual decrease in the distance between the eye and the shifted target position and also the adaptation-induced changes in the perceived location of objects (Masselink & Lappe, 2021). More specifically, the mislocalization is assumed to develop because the computed displacement of visual space underestimates the adaptive changes to the performed saccade vector (Masselink & Lappe, 2021).

The characteristics of oculomotor learning following subtle intra-saccadic manipulations are well studied, but what about oculomotor learning following obvious intra-saccadic manipulations? Two recent studies demonstrate that oculomotor learning following obvious intra-saccadic manipulations is possible: Heins and Lappe (2024) demonstrated that participants are capable of adjusting their gaze to achieve task success based on artificial feedback signals that were added to the post-saccadic scene. In addition, Heins and Lappe (2022b) demonstrated that oculomotor learning develops following obvious color swaps in adjacent objects that form an object array. The learning curves resembled those observed for saccade adaptation following subtle intra-saccadic displacements of the entire object array. However, although the overt oculomotor behavior appears to be similar following subtle and obvious intra-saccadic manipulations, the results leave open the question of whether the error signals and mechanisms involved in both learning processes are the same. To address this question, we combine the experimental paradigm developed in Heins and Lappe (2022b) with a localization task. This specific paradigm allows the comparison of oculomotor learning following subtle and obvious intra-saccadic manipulations, and even a combination of both. In separate recording sessions, three different intra-saccadic manipulations are applied: 1) a subtle intra-saccadic shift of the object array against saccade direction, 2) an obvious color swap against saccade direction in adjacent objects, and 3) a combination of a subtle intra-saccadic shift of the object array against saccade direction with an obvious color swap in saccade direction. In addition, the paradigm is suitable to study the consequences of the different manipulations of the visual stimulus independent from the executed saccades, thereby providing the opportunity to disentangle the impact of the intra-saccadic manipulation and the motor behavior on spatial perception. This aspect is particularly interesting given that adaptive changes to the overt oculomotor behavior and the perceived location of objects are not necessarily aligned (Heins & Lappe, 2022a; Tyralla, Pomè, & Zimmermann, 2023), indicating that different processes or error signals might contribute to saccade targeting and spatial perception.

We hypothesize that 1) the subtle intra-saccadic displacement of the object array will induce a localization shift in the direction of the array shift, consistent with the findings for intra-saccadic displacements of isolated target objects. For the obvious color swap, we expect 2) no shift in perceived location. We assume that the cause of the post-saccadic error will be attributed to the clearly visible change in the external world during the eye movement rather than to erroneous motor performance, thus preventing the process of postdicting and nullifying a motor error. Consequently, when both manipulations are combined, we hypothesize that 3) a localization shift consistent with the direction of the array shift will develop. We posit that the obvious color swap will be identified as a change in the external world, leaving the stepped array position as the sole credible source of information for calculating the postdicted motor error. Finally, we expect that 4) although the overt oculomotor behavior will depend on the combination of intra-saccadic manipulation and instruction, the object localization will be affected by the intra-saccadic manipulation only.

## Experiment 1

### Method

#### Sample

The sample consisted of 24 participants (22 female). The participants were between 19 and 46 years of age (*M* = 22.92, *SD* = 6.64) and had normal or corrected-to-normal vision. Each participant gave written informed consent at the beginning of the first recording session. As a compensation for their time, the participants either received course credit or 8 €/h.

#### Experimental setup

The experiment was carried out in the Institute for Psychology of the Universität Münster. The room was dimly lit and participants were seated with a distance of 67 cm in front of an Eizo FlexScan 22-inch monitor (Eizo, Hakusan, Japan). The participants placed their head on a chin–forehead rest to minimize head movements during the experiment. The frame rate of the monitor was 75 Hz and the screen resolution was 1152 × 870 pixels. An Eyelink 1000 eye tracker (SR Research, Ontario, Canada) with a sampling rate of 1,000 Hz was used to record the eye movements. Viewing was binocular, although tracking was restricted to the right eye. The code for running the experiment was written in MATLAB (Mathworks, Natick, MA). The Psychophysics Toolbox extension (Brainard, 1997; Kleiner et al., 2007) was used for stimulus presentation. The experimental procedure was approved by the Ethics Committee of the Faculty of Psychology and Sports Science at the University of Münster.

#### Stimuli and procedure

The stimuli were displayed on a light gray background. Each trial began with the presentation of a red fixation cross (0.6° × 0.6°) on the screen. Its vertical anchor position was the center of the screen and its horizontal anchor was 8° to the left of the screen center. Noise was added to this position in a counterbalanced manner on both the vertical (−1°, 0°, 1°) and the horizontal (−2, −1, 0°, 1°, 2°) axis. Participants had to look at the fixation cross for at least 300 ms to establish an initial stable fixation. Once the initial stable fixation had been confirmed, three geometric figures appeared to the right of the fixation cross: a circle (diameter: 1°), an isosceles triangle (base = 1°, height = 1°), and a square (width = 1°). The centers of the objects were located 8°, 10°, and 12° to the right of the fixation cross, each 2° apart. The three objects formed an object array. Their order within this array varied from trial to trial. In each trial, one of the objects was colored red and the other objects were dark gray. The participants continued to hold fixation for another 500 to 1,000 ms until the fixation cross was removed. If the eye position left the fixation window (an area of 4° × 4° extending around the fixation cross) before the fixation cross was removed, a sine tone indicated that the fixation had been interrupted prematurely. In that case, the current trial was aborted and restarted. Removing the fixation cross served as the go-signal for the saccade. Subsequent events depended on the type of trial and the intra-saccadic manipulation.

#### Conditions

We used instructions that were similar as in Heins and Lappe (2022b). The instruction “color” prompted the participants to always look at the colored object within the object array. In case they did not foveate the colored object after their primary saccade, the participants should correct their gaze toward the colored object. The instruction “shape” prompted the participants to always look at the object with the shape that was indicated by the red color before the primary saccade. For example, if the triangle in the array was colored red during the pre-saccadic fixation period, then the participants were to direct their gaze toward the triangle. In case they did not foveate the triangle after their primary saccade, gaze should be corrected toward the triangle. Participants pseudorandomly received one of these instructions. All participants performed three recording sessions of approximately 25 minutes each during which the different intra-saccadic manipulations were applied. The time interval between recording sessions was set to a minimum of 72 hours.

We used the same intra-saccadic manipulations as in Heins and Lappe (2022b). In the array shift condition (AS), the entire object array was shifted 2° to the left upon saccade onset, which was detected once the eye had reached a velocity of 100 deg/s and had travelled 3° toward the target object. This criterion ensured that the intra-saccadic manipulation was applied before saccade offset (color instruction = 95.60%; shape instruction = 95.97%). The participants had to shorten the saccade amplitude to foveate the target object, regardless of the instruction type. The different information sources within the post-saccadic image were congruent.

In the color swap condition, the object array maintained its position but a color swap occurred. The object that was red initially turned gray upon detection of saccade onset, and, simultaneously, the object one position further inward became red. This color change induced a conflict between different feature dimensions of the target object. For the participants who received the color instruction, the target-defining feature dimension was color. Consequently, they had to decrease the saccade gain to foveate the red object and, in doing so, achieved task success despite incongruent shape information. For the participants who received the shape instruction, the target-defining feature dimension was the shape of the object. Thus, they had to maintain a stable saccade gain to comply with the instruction despite the incongruent color information. The color swap reliably occurred before saccade offset (color instruction − 98.94%; shape instruction = 99.35%).

**Figure 1.**
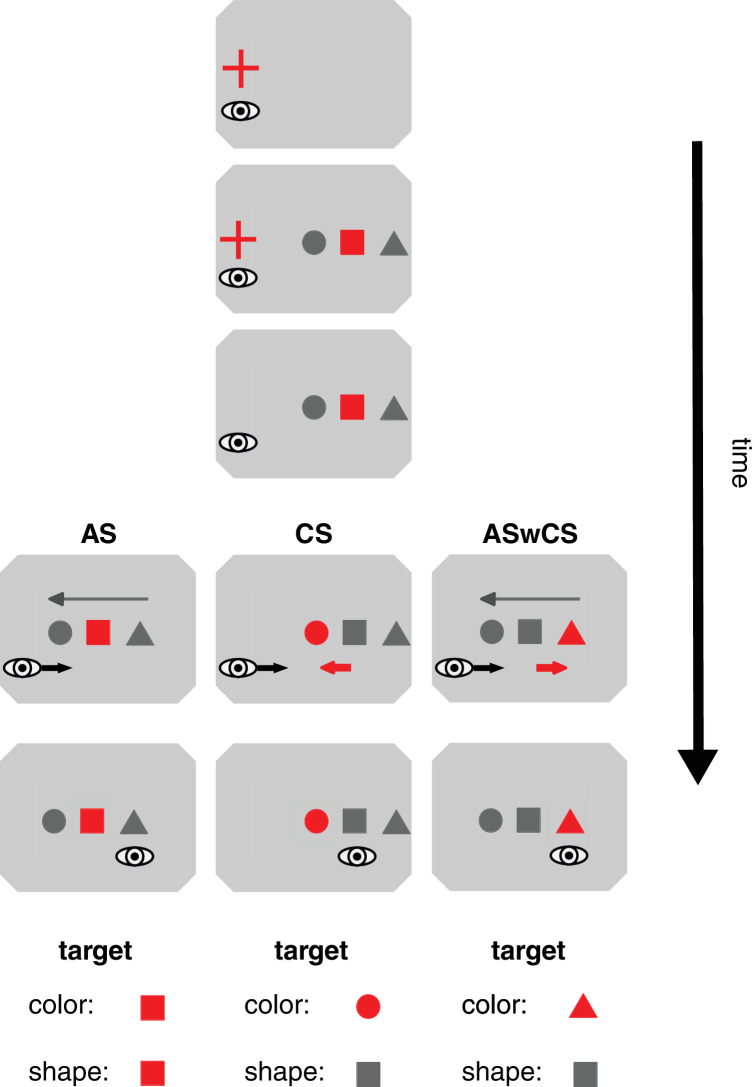
The three different intra-saccadic manipulations and the corresponding post-saccadic images. The participants looked at the fixation cross and continued to hold fixation when the object array was drawn to the screen. In the AS condition, the entire object array was shifted 2° to the left upon saccade onset. The post-saccadic information unambiguously indicated that the saccade overshot the target. This was equally true for the color and shape instruction. In the CS condition, the object to the left of the initially colored object turned red as soon as saccade onset was detected. The post-saccadic information regarding the target object was ambiguous. For the color instruction, the saccade gain needed to be shortened to land on target. For the shape instruction, the saccade gain had to be kept stable to foveate the task-defined target. In the ASwCS condition, the object array shifted 2° to the left upon saccade onset and simultaneously, the object to the right of the initially colored object turned red, leading to ambiguous feedback regarding the target object. Following the color instruction, the saccade gain had to be kept stable. Following the shape instruction, the saccade gain had to decrease for the eye to land on the instruction-defined target. The eye icon in the bottom row indicates the expected saccade end position at the beginning of the adaptation phase. Stimuli are not drawn true to scale.

In the AS with color swap condition (ASwCS), the object array was shifted 2° to the left once saccade onset was detected. A color swap occurred simultaneously: the object to the right of the initially colored object became red. This manipulation also induced conflict regarding the target feature dimensions. The participants who received the color instruction had to maintain a stable saccade gain to foveate the red object. They had to ignore the incongruent shape information. The participants who received the shape instruction had to decrease their saccade gain to foveate the object with the correct shape but had to ignore the incongruent color information. The combined intra-saccadic manipulation was applied while the eye was still in flight in most cases (color instruction = 97.64%; shape instruction = 94.91%). Figure 1 illustrates the sequence of events for all three intra-saccadic manipulations and indicates the expected early-adaptation saccade landing position as well as the instruction-defined post-saccadic target object.

**Figure 2.**
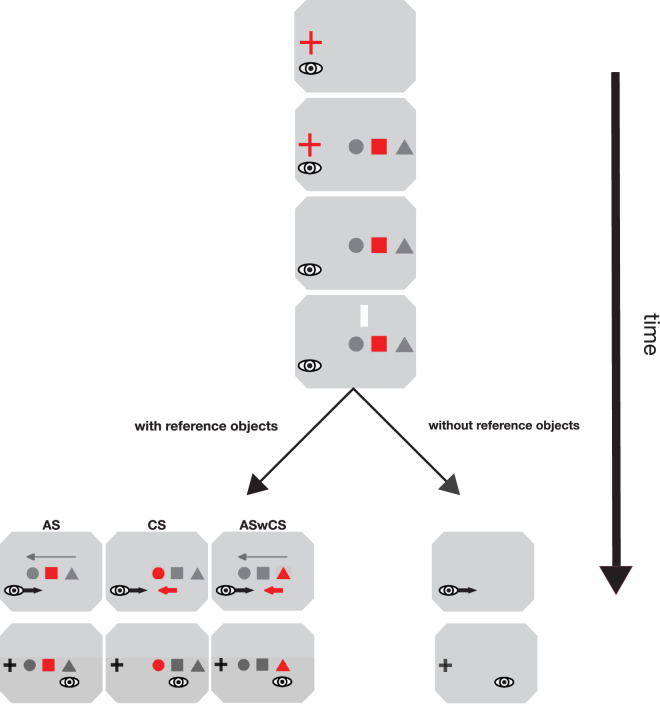
The localization task before and after the learning phase. Participants looked at the fixation cross and continued to hold fixation when the object array appeared on the computer screen. The fixation cross was removed. After 50 ms, a white rectangle was presented. This timing ensured that the localization stimulus was shown during the saccade preparation period. Once saccade onset was detected, the object array was either removed (localization without reference objects), or the intra-saccadic manipulation was applied (localization with reference objects). In all localization trials, the cursor was displayed on screen 750 ms after the saccade was detected. The participants moved it to the perceived location of the white rectangle. The eye position that is indicated in the bottom row corresponds with the expected saccade end position at the beginning of the adaptation phase. Stimuli are not drawn true to scale.

All experimental sessions began with 40 localization trials. The participants looked at the fixation marker until it disappeared. At 50 ms after the fixation cross was removed, a white rectangle (1° × 2°) was presented for a maximum duration of 28 ms (2 frames). The mean presentation duration of the localization stimulus was 14.37 ms across all conditions. The vertical position of the rectangle was always 3° above the fixation cross. Its horizontal position corresponded to one of the three objects, but additional noise (−1°, 0°, 1°) was added. During one-half of the localization trials, the object array was removed from the screen following the detection of saccade onset. During the other half, the intra-saccadic manipulation was applied upon saccade onset. At 750 ms after the rectangle was presented, the cursor appeared at the former position of the fixation cross. The cursor was black and shaped like a crosshair. It remained visible while participants moved the cursor to the perceived location of the rectangle. Once a button press was registered, the current cursor position was recorded as the localization judgment and the cursor was immediately removed from the screen. The perceived location of the localization stimulus was thus measured in the absence and in the presence of reference objects (Figure 2). The two types of localization trials were presented alternately. Participants were instructed to click in the lower right screen corner if they did not perceive the localization stimulus at all. The mean flash latency, that is, the mean time interval between the onset of the rectangle and onset of the saccadic eye movement, indicated that the localization stimulus was presented during the saccade preparation period and well before saccade onset. Localization trials during which the flash latency was below 100 ms were excluded from the data analysis to make sure that the results were not affected by the compression effect (Georg & Lappe, 2009; Lappe, Awater, & Krekelberg, 2000; Ross, Morrone, & Burr, 1997). In the AS condition, the mean flash latency was 214.52 ms (*SD* = 65.87) for the shape instruction and 220.91 ms (*SD* = 92.79) for the color instruction. In the CS condition, the average flash latency was 195.32 ms (*SD* = 50.02) for the shape and 211.92 ms (*SD* = 66.68) for the color instruction. In the ASwCS condition, the average flash latency was 219.00 ms (*SD* = 97.98) for the shape and 203.06 ms (*SD* = 49.43) for the color instruction.

#### Data analysis

To include only those trials during which the oculomotor behavior was correctly captured and participants followed the task, we defined a set of inclusion criteria for primary saccades. The saccade amplitude had to be larger than 4° and smaller than 16°. The duration had to be less than 100 ms and the minimum latency was set to 100 ms after the fixation cross was removed. After applying these criteria, 91.51%, 91.20%, and 89.66% of primary saccades were included in the AS, CS, and ASwCS conditions, respectively. Localization judgments were included if the onset of the localization stimulus preceded the saccade onset by more than 100 ms and if the localization stimulus was presented for 28 ms or less. Localization judgments that were either closer than 4° to the lower screen border or closer than 2° to the right screen border were excluded to avoid the inclusion of trials during which participants did not see the localization stimulus. In addition, the saccade in the respective localization trial had to be valid for the localization judgment to be included. These criteria were met for 83.39%, 88.23%, and 87.29% of localization judgments in the AS, CS, and ASwCS conditions, respectively. We assessed the saccade gain *G* throughout the learning procedure instead of the saccade amplitude. This was done because the participants made saccades to the different object locations within the array and each of the three corresponds with a different required amplitude. The saccade gain quantifies the ratio of the actual saccade amplitude and the distance the eye needed to travel to reach the target object (i.e., the distance between fixation cross and pre-saccadically colored object). To quantify the adaptation magnitude, we then calculated saccade gain change *GC*. In this equation, ${\bar{G}}_{early}$ corresponds with the average saccade gain during the first 20 adaptation trials that followed the localization trials.

(1) $$\begin{array}{c} GC=\frac{G-{\bar{G}}_{early}}{{\bar{G}}_{early}}\times 100.\; \end{array}$$

We computed a mixed ANOVA on the localization change. We assessed the influence of the between-subjects factor instruction (shape, color) and the within-subjects factors feedback (AS, CS, ASwCS) and presence of reference objects (present, absent) on the localization shift that developed from pre- to post-adaptation. We tested if the residuals of the model followed a normal distribution and checked the assumptions of sphericity and homoscedasticity. A moderate deviation from the normal distribution was detected. We therefore computed a permutation analysis of variance (ANOVA) (Kherad-Pajouh & Renaud, 2015) (100,000 permutations) with the permuco package (Frossard & Renaud, 2021) instead of a parametric ANOVA. We calculated the contrasts for our initial hypotheses following the ANOVA using the emmeans-package (Lenth, 2025) and additionally tested the localization change in the different conditions against zero. If the assumption of normality was violated, Wilcoxon-signed-rank tests were calculated instead of *t*-tests. The alpha-level of the tests was adjusted using the Bonferroni–Holm procedure. Following this analysis, we calculated the average within-participant dispersion per combination of manipulation and instruction, separately for trials in which the localization judgment was made with or without reference objects. We chose the mean absolute deviation as a measure of within- subject dispersion. It describes the average absolute distance of each data point from the participant mean. This concept weighs individual outliers less heavily than the standard deviation, because it is based on the absolute distance from the mean rather than the squared distance.

We also computed an explorative mixed analysis of variance on the localization error. We investigated the influence of the factors instruction, manipulation, and experiment phases (early, late) on the magnitude of the localization error. The Greenhouse–Geisser correction was applied because the assumption of sphericity was violated. Only meaningful comparisons are reported, but the alpha level was corrected for the entire family of tests using the Bonferroni–Holm procedure. As effect size measures, we report partial eta squared and Cohen’s *d*.

For the correlation between localization change and saccade gain change, each participants’ average localization change during the post-adaptation localization trials and the average saccade gain change during the last 20 adaptation trials were included. Kendall’s tau was used because of the small sample size and because the data deviated from the normal distribution in some experimental conditions.

For data preprocessing, MATLAB (version R2024a) was used. The data analysis and visualization was conducted in R (version 4.4.2; The R Foundation for Statistical Computing, Vienna, Austria).

### Results

#### Gain change

**Figure 3.**
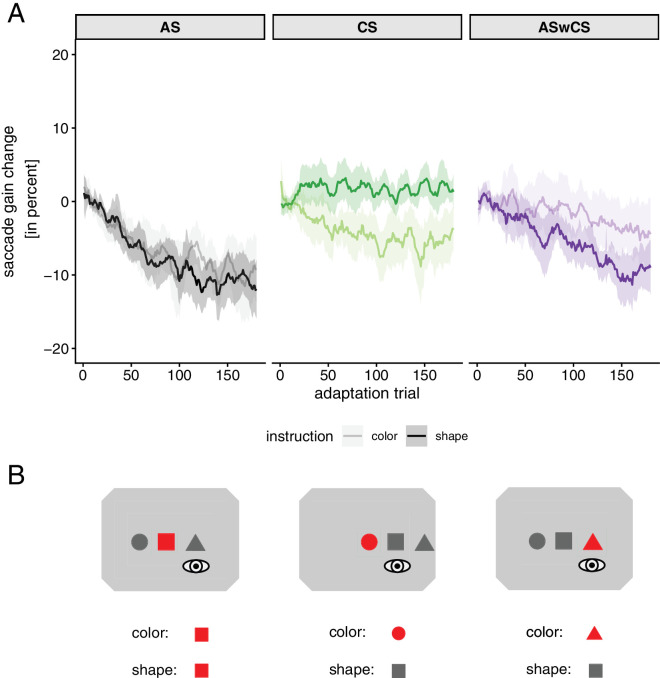
(A) Moving average of the saccade gain change (window size = 10 trials) throughout the adaptation phase of the experiment. The learning curves for the color instruction (lighter shade) and the shape instruction (darker shade) are plotted with the corresponding 95% confidence intervals (shaded area). The data are plotted separately for the manipulations AS, CS, and ASwCS. (B) A typical post-saccadic image for each manipulation. The eye icon illustrates the saccade landing position before adaptation. The instruction-defined target object is indicated below.

We observed the saccade gain change throughout the learning phase following the instruction color (Figure 3A) and following the instruction shape (Figure 3B). Following the instruction color, the saccade gain decreased gradually in the AS condition, which served as the control condition because of the unambiguous post-saccadic feedback. In the CS condition, the saccade gain decreased in a similar manner as the participants shortened their saccade amplitude to foveate the red object. However, the gain change appears to be less pronounced than in the AS condition, possibly indicating interference from the incongruent shape information. In the ASwCS condition, the saccade gain change was even weaker, which indicates compliance with the instruction because looking at the red object requires a stable saccade gain. Following the instruction shape, the saccade gain in the AS condition decreased continuously until the end of the learning phase. In the ASwCS condition, the saccade gain change follows a similar learning curve, indicating that the participants shortened the saccade amplitude to look at the object with the correct shape. In contrast, in the CS condition, a stable saccade gain was maintained to foveate the object with the correct shape, indicating complience with the task.

These results largely replicate the results of our previous study on the task-dependent use of post-saccadic information (Heins & Lappe, 2022b) although some differences are apparent. First, following the color instruction, the saccade gain in the CS condition does not decrease in parallel to the control condition. Second, also following the color instruction but now in the ASwCS condition, the saccade gain does not appear to be entirely unaffected by the task-irrelevant color swap. This pattern of results deviates slightly from the results we observed in the original study, which indicated no interference from the incongruent information source during the learning phase. Two reasons could account for these differences. 1) The trial structure of the original and the current experiments differ slightly because the learning phase of the original study consisted of 200 trials compared with 180 trials in the current study. 2) Each session of the original study began and ended with 20 trials during which the object array was removed as soon as saccade onset was detected. The oculomotor behavior during these trials was used to determine a baseline against which adaptive changes to the saccade metrics were quantified. These baseline trials were replaced with the localization task that was measured before and after the learning procedure. The current study still confirms the original study’s main result: information sources within the post-saccadic image are used flexibly, depending on the current task. The error evaluation depends on the selected target object which is defined by the task. Therefore, different patterns of oculomotor learning can develop despite identical intra-saccadic manipulations (Heins & Lappe, 2022b).

#### Localization change

**Figure 4.**
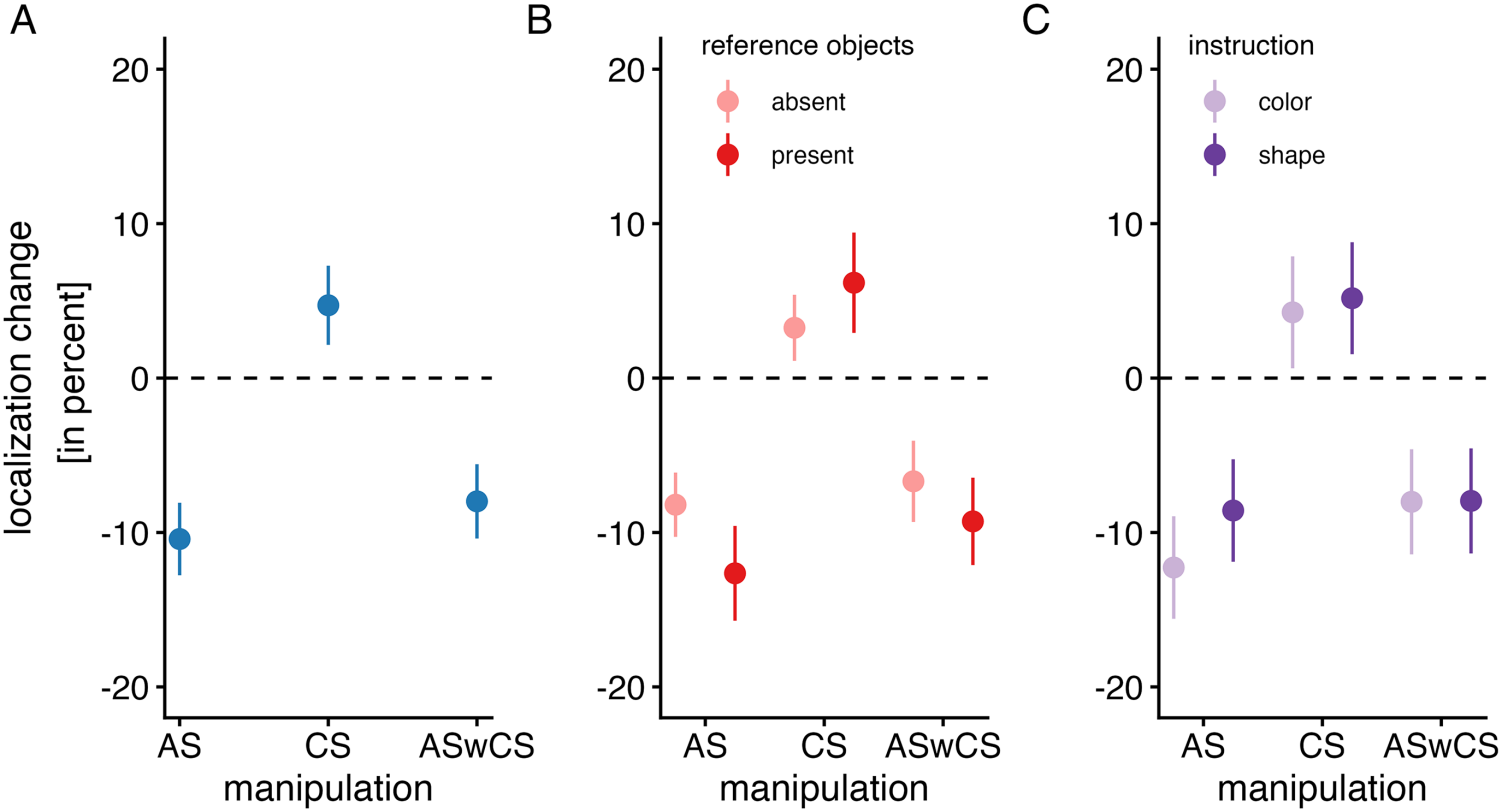
Localization change. The dots illustrate the estimated marginal means and the corresponding 95 percent confidence interval for (A) the main effect of manipulation, (B) the interaction of manipulation and the presence of post-saccadic reference objects, and (C) the interaction of manipulation and instruction.

After we confirmed that different instructions lead to different patterns of oculomotor learning despite the same intra-saccadic manipulation, we investigated how the interplay of instruction, that is, task, and intra-saccadic manipulation affected the development of the shift in perceived object location. We computed a mixed ANOVA with the between factor instruction (shape, color) and the within factors manipulation (AS, CS, and ASwCS) and the presence of reference objects (present, not present). The main effects of instruction, *F*(1,22) = 0.69, *p* = 0.415, $\eta_{p}^{2}$ = 0.03, and object presence, *F*(1,22) = 3.09, *p* = 0.093, $\eta_{p}^{2}$ = 0.12, were not significant and neither were the interactions between instruction and manipulation, *F*(2,44) = 1.18, *p* = 0.316, $\eta_{p}^{2}$ = 0.05 (Figure 4C); instruction and object presence, *F*(1,22) = 0.02, *p* = 0.901, $\eta_{p}^{2}$ = 0.001; and instruction, manipulation and object presence, *F*(2,44) = 0.27, *p* = 0.765, $\eta_{p}^{2}$ = 0.01. However, the main effect of manipulation, *F*(2,44) = 86.04, *p* < 0.001, $\eta_{p}^{2}$ = 0.80, was significant, indicating that the type of intra-saccadic manipulation influenced the localization change of objects (Figure 4A). In the AS and ASwCS conditions, a localization shift of similar magnitude developed, *t*(22) = −1.89, *p* = 0.072, *d* = −0.40, consistent with the direction of the AS. In the CS condition, the perceived location of the localization stimulus shifted in outward direction and, thus, the localization change developed in opposite direction. Congruently, the localization change in the CS condition was different than in the AS condition, *t*(22) = −16.30, *p* < 0.001, *d* = −3.47, and the ASwCS condition, *t*(22) = 8.81, *p* < 0.001, *d* = 1.88. In addition, the interaction of manipulation and object presence, *F*(2,44) = 15.24, *p* < 0.001, $\eta_{p}^{2}$ = 0.41, was significant, indicating that the presence of reference objects might affect the localization change differently in the different manipulation conditions (Figure 4B). The localization change was more pronounced when the localization judgment was given in the presence of reference objects. This difference was significant for all manipulation conditions, AS: *t*(22) = 3.94, *p* < 0.001 *d* = 0.84; CS: *t*(22) = −3.03, *p* = 0.006, *d* = −0.65; ASwCS: *t*(22) = 2.08, *p* = 0.049, *d* = 0.44.

Finally, we tested whether the localization change that occurred was significant. For the AS condition, the localization change in direction of the array shift was significant both in the presence (*z* = −4.93, *p* < 0.001, *d* = −2.01) and in the absence of reference objects, *t*(23) = −7.77, *p* < 0.001, *d* = −3.24. The same is true for the ASwCS condition: present: *t*(23) = −6.96, *p* < 0.001, *d* = −2.90; absent: *t*(23) = −5.39, *p* < 0.001, *d* = −2.25. However, in the CS condition, significant localization change developed against the direction of the color swap, present: *z* = −4.04, *p* < 0.001, *d* = −1.65; absent: *z* = −3.26, *p* = 0.001, *d* = −1.33. In Figure 4, the estimated marginal means are plotted with their 95% confidence interval. Observed means and standard deviations are included in Table 1.

**Table 1.** Localization change from pre- to post-learning. Means and standard deviations in percent.

	Color				Shape			
	Absent		Present		Absent		Present	
	*M*	*SD*	*M*	*SD*	*M*	*SD*	*M*	*SD*
AS	−10.07	5.00	−14.47	7.53	−6.34	6.96	−10.81	6.96
CS	3.13	4.57	5.39	6.67	3.38	8.55	6.96	8.55
ASwCS	−6.88	5.78	−9.15	7.10	−6.49	6.23	−9.42	6.23

Previous studies have demonstrated that the gain change of a saccade and the localization change correlate (Collins et al., 2007; Schnier et al., 2010). One might expect this correlation to emerge in our congruent conditions (during which the saccade gain change and the localization change develop in the same direction) and to be absent in the incongruent conditions. However, this correlation only occurs reliably if the gain change of a saccade to a given position and the localization change for a localization stimulus at that same position are taken into account (Collins et al., 2007; Heins & Lappe, 2022a; Schnier et al., 2010). In our study, by contrast, both the location of the saccade target and the localization stimulus varied considerably across trials. Correspondingly, when correlating the saccade gain change and the localization change, no significant relationship was found in any experimental condition (all *p* > 0.250).

The average within-subject dispersion of the localization change in AS condition and following the color instruction was 13.42% with and 11.14% without post-saccadic reference objects (shape: with reference objects, 12.84%; without reference objects, 9.95%). In the CS condition, the average within-subject dispersion following the color instruction was 14.21% with and 11.00% without reference objects (shape: with reference objects, 11.03%; without reference objects, 10.37%). In the ASwCS condition, the dispersion following the color instruction was 11.47% with and 9.28% without reference objects (shape: with reference objects, 10.87%; without reference objects, 10.35%).

#### Localization error

The localization change in the outward direction observed in condition CS was unexpected. To better understand potential causes of this localization shift, we computed an additional mixed ANOVA with the within factors phase (early, late) and manipulation (AS, CS, ASwCS) and the between factor instruction (color, shape). The main effects of phase, *F*(1,22) = 37.06, *p* < 0.001, $\eta_{p}^{2}$ = 0.63, and manipulation, *F*(1.56,34.29) = 22.62, *p* < 0.001, $\eta_{p}^{2}$ = 0.51, were significant, but the main effect of instruction was not, *F*(1,22) = 1.01, *p* = 0.325, $\eta_{p}^{2}$ = 0.04. The interactions of manipulation and phase, *F*(2,44) = 76.91, *p* < 0.001, $\eta_{p}^{2}$ = 0.78, and manipulation and instruction, *F*(1.56,34.29) = 3.78, *p* = 0.043, $\eta_{p}^{2}$ = 0.15, were both significant, but the interaction of phase and instruction, *F*(1,22) = 1.00, *p* = 0.328, $\eta_{p}^{2}$ = 0.04, as well as the three-way interaction of manipulation, phase and instruction, *F*(2,44) = 1.64, *p* = 0.206, $\eta_{p}^{2}$ = 0.07, was not.

The post hoc analysis confirmed that the localization error in condition AS was different from the localization error in the CS condition, *t*(22) = −6.92, *p* < 0.001, *d* = −1.47, and the ASwCS condition, *t*(22) = −4.76, *p* < 0.001, *d* = −1.02. In addition, the localization error in the CS and ASwCS conditions was different, *t*(22) = 2.56, *p* = 0.018, *d* = 0.55. Following the color instruction, the localization error in the AS condition was different than in the CS condition, *t*(22) = −3.32, *p* = 0.040, *d* = −0.71, and the ASwCS condition, *t*(22) = −3.74, *p* = 0.016, *d* = −0.80. The difference between the CS condition and the ASwCS condition was not significant, *t*(22) = 0.33, *p* = 1.00, *d* = 0.07. Following the shape instruction, the localization error in AS was different than in the CS condition, *t*(22) = −6.46, *p* < 0.001, *d* = −1.38, but not different from the localization error in the ASwCS condition, *t*(22) = −2.99, *p* = 0.074, *d* = −0.64. The localization error in the CS condition was different than in the ASwCS condition, *t*(22) = 3.30, *p* = 0.040, *d* = 0.70.

At the beginning of the experiment, there was no significant difference in the mean localization error between the AS and CS conditions, *t*(22) = 2.13, *p* = 0.180, *d* = 0.45, or the AS and ASwCS conditions, *t*(22) = −1.71, *p* = 0.307, *d* = −0.36. However, the localization error in the CS and ASwCS conditions was different at the beginning of the experiment, *t*(22) = −3.18, *p* = 0.021, *d* = −0.68.

**Figure 5.**
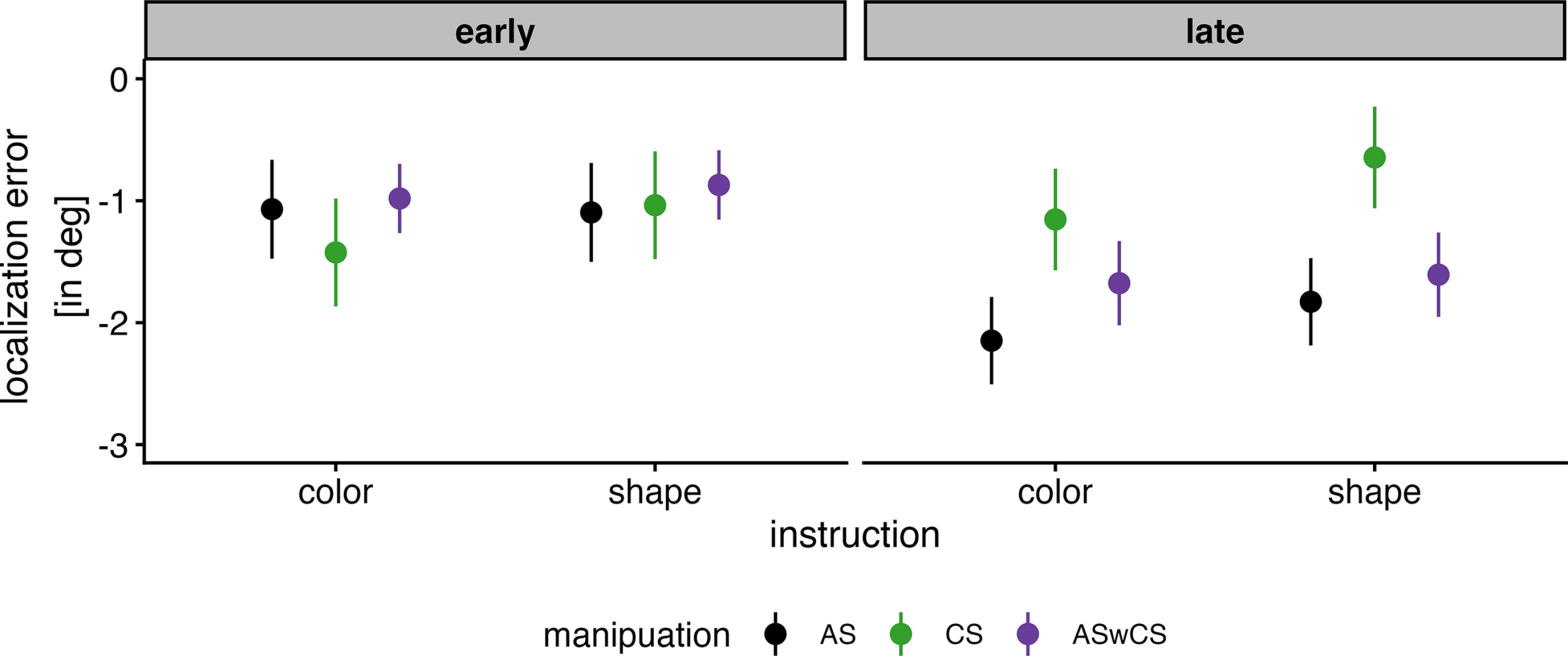
Localization error, depicted separately for the early and late localization judgments. The dots illustrate the estimated marginal means and the corresponding 95% confidence interval.

The localization error changed significantly from the beginning to the end of the experiment in all conditions, AS: *t*(22) = 9.21, *p* < 0.001, *d* = 1.96; CS: *t*(22) = −4.13, *p* = 0.003, *d* = 0.88; ASwCS: *t*(22) = 7.01, *p* < 0.001, *d* = 1.49. However, while the localization error increased in the AS and ASwCS conditions, it became smaller in CS condition and approached zero (Figure 5). Congruently, the localization error at the end of the experiment was different in the AS and CS conditions, *t*(22) = −12.53, *p* < 0.001, *d* = −2.67, as well as in the CS and ASwCS conditions, *t*(22) = 6.58, *p* < 0.001, *d* = 1.40. Yet, the localization error in the AS and ASwCS conditions was also different, *t*(22) = −5.47, *p* < 0.001, *d* = −1.17, at the end of the experiment.

In Figure 5, the estimated marginal means are plotted with their 95% confidence intervals. Observed means and standard deviations are included in Table 2.

**Table 2.** Localization error at the beginning and at the end of the experiment. Means and standard deviations in degrees.

	Color				Shape			
	Early		Late		Early		Late	
	*M*	*SD*	*M*	*SD*	*M*	*SD*	*M*	*SD*
AS	−1.07	0.72	−2.15	0.44	−1.10	0.63	−1.83	0.72
CS	−1.42	0.77	−1.15	0.59	−1.04	0.71	−0.65	0.79
ASwCS	−0.98	0.40	−1.68	0.52	−0.87	0.54	−1.61	0.63

### Interim discussion

For all three manipulations, participants underestimated the eccentricity of the localization stimulus at the beginning of the experiment. For the AS and ASwCS conditions, during which the array shifted against saccade direction, the localization error increased from pre- to post-adaptation because participants demonstrated the expected shift in perceived location in inward direction. In the CS condition, however, participants localized the localization stimulus closer to its actual position, leading to a decrease in localization error and to the localization shift in outward direction. Following the analysis of the localization error, we hypothesized that the localization error might have decreased because participants became more familiar with the intra-saccadic manipulation and the localization task itself. This increased familiarity might explain the localization shift in outward direction. To rule out other reasons for the unexpected localization shift in outward direction, we conducted two additional experiments.

## Experiments 2 and 3

During both experiments, we used three identical squares to form the object array. We used the intra-saccadic manipulation CS only, but applied it both in inward (Experiment 2) and outward (Experiment 3) directions. Because the main effect of instruction on localization change was not significant in Experiment 1, we only used the color instruction.

The objective of the additional experiments was to investigate the perceptual consequences of the color swap without the influence of object shape or identity. If the color swap in inward direction still leads to a localization shift in an outward direction, then it develops independently of the shape or identity of the objects within the array. In addition, applying the color swap in an outward direction (Experiment 3) is a direct test if the color swap itself causes the localization shift observed in Experiment 1. If the color swap in the outward direction leads to a localization shift in an outward direction as well, then the localization shift develops regardless of the direction of the color swap. This result would strengthen our hypothesis that the decrease in localization error from pre- to post-adaptation, that was observed in Experiment 1, developed indeed due to practice or familiarity.

### Methods

#### Sample

The sample for Experiment 2 consisted of 12 participants (8 female). The age range was 19 to 41 years (*M* = 24.42, *SD* = 7.09). The sample for Experiment 3 consisted of 13 participants (10 female). Their age ranged between 19 and 37 years (*M* = 21.69, *SD* = 4.89). One participant had to be excluded from the data analysis because the inclusion criteria were violated in more than 50% of the trials.

If a participant took part in both Experiments 2 and 3, a minimum of 72 hours had to pass between the recording sessions. Eight participants participated in both experiments. As in Experiment 1, all participants had normal or corrected-to-normal vision and gave written informed consent. As a compensation for their time, the participants received either course credit or 8 €/h.

#### Experimental setup

The experimental setup was the same as in Experiment 1.

### Stimuli and procedure

The stimuli and procedure were similar to that in Experiment 1. All participants were instructed to look at the colored square within the object array. In case they did not foveate the colored square after their primary saccade, the participants should correct their gaze toward the red square. As in Experiment 1, the object array appeared once the initial stable fixation had been confirmed. The three squares appeared to the right of the fixation cross (width = 1°). The centers of the squares were 8°, 10°, and 12° to the right of the fixation cross, respectively. In each trial, one of the squares was red and the other objects were dark gray. The participants continued to hold fixation until the fixation cross was removed. Subsequent events depended on the type of trial.

During the adaptation trials, the object array remained stationary but a color swap occurred once saccade onset was detected. In Experiment 2, the square that was red initially turned gray upon detection of saccade onset and, simultaneously, the square to the left became red. Consequently, the participants had to decrease the saccade gain to foveate the red object. In Experiment 3, the square that was red before the saccade turned gray upon saccade onset and the square to the right turned red. Thus, participants had to increase the saccade gain to foveate the red object.

The localization trials were the same as in Experiment 1. Localization trials with and without post-saccadic reference objects were presented alternately. The average flash latency was 208.42 ms (*SD* = 57.19) in Experiment 2 and 189.06 ms (*SD* = 39.28) in Experiment 3.

#### Data analysis

The inclusion criteria were the same as in Experiment 1. In Experiment 2, 92.63% of the primary saccades and 86.77% of the localization trials were included in the data analysis. In Experiment 3, 92.69% of the primary saccades and 87.71% of the localization trials were valid. Saccade gain change and localization change were computed equivalently to Experiment 1. We used *t*-tests to test whether 1) the localization change deviates from zero and 2) if the localization change is different between Experiments 2 and 3. Additional *t*-tests were computed to investigate if the localization error decreases from pre- to post-adaptation. If the variances were unequal, a Welch- *t*-test was computed instead of a conventional *t*-test. In case the data were not normally distributed, Wilcoxon signed-rank tests were used. As in Experiment 1, the Bonferroni–Holm procedure was applied to control for the inflation of the alpha level.

### Results

#### Saccade gain change

**Figure 6.**
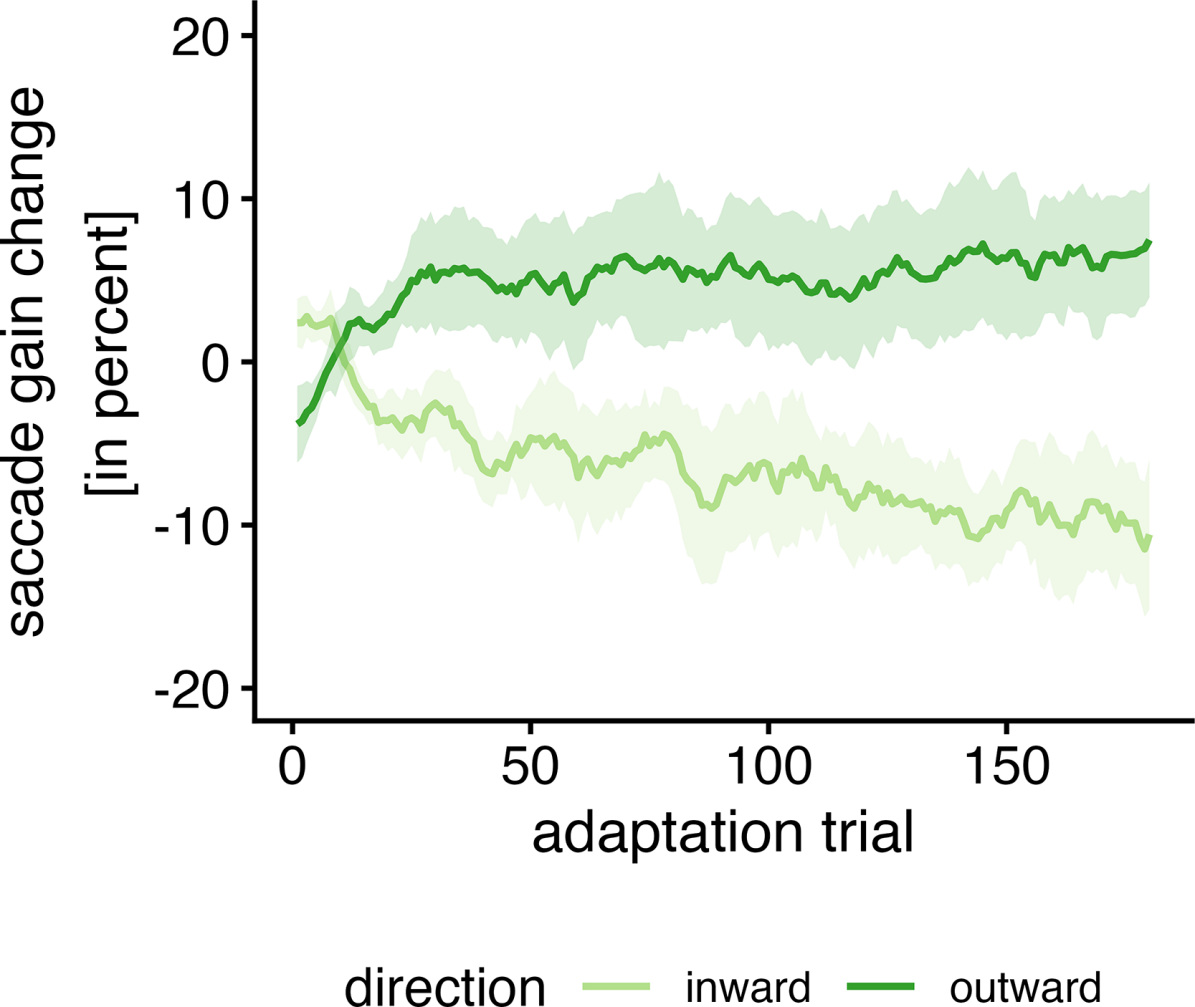
Saccade gain change throughout the learning phase of Experiment 2 (inward color swap) and 3 (outward color swap).

We investigated the saccade gain change throughout the learning phase of Experiments 2 and 3 (Figure 6).

Participants in both experiments were instructed to look at the colored object. In Experiment 2, following the color swap in an inward direction, the saccade gain decreased throughout the learning phase. This decrease in saccade gain is consistent with the results from Experiment 1, and it indicates that participants decreased their saccade gain to foveate the red object. In Experiment 3, following the color swap in an outward direction, the saccade gain increased throughout the learning phase, indicating that participants made longer saccades to look at the colored object. This pattern of results demonstrates that the amplitude of the primary saccade can also be lengthened if that is required to comply with the current task.

#### Localization change

**Figure 7.**
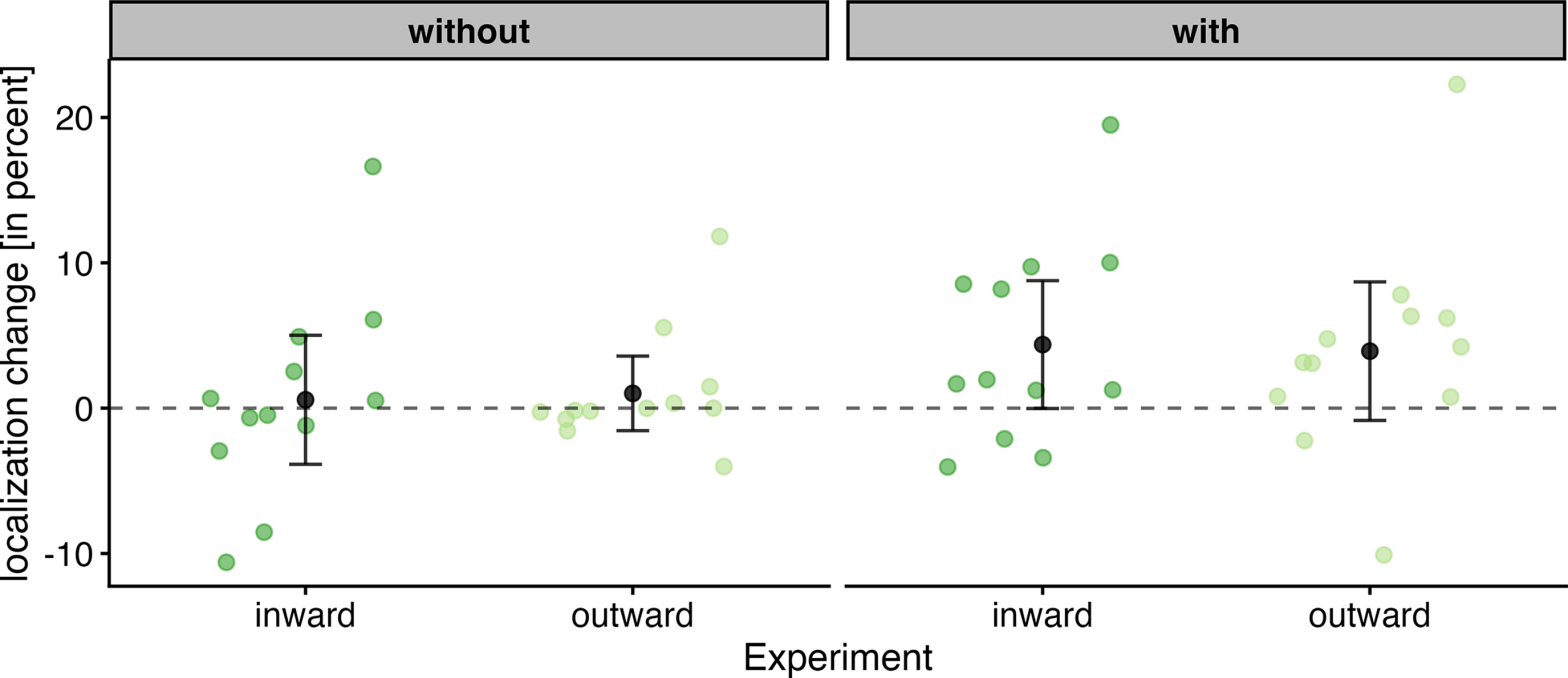
Localization change in Experiments 2 (inward color swap) and 3 (outward color swap). The black dots illustrate the average localization change in the respective experiment, depicted separately for localization judgments made in the absence or presence of post-saccadic reference objects. The bars depict the corresponding 95% confidence interval. The green dots illustrate the average localization change of each participant.

We aimed to determine whether the intra-saccadic color swap impacts the perceived location of objects or if increased familiarity with the intra-saccadic manipulation or the localization task causes the reduction of the localization error and, therefore, the localization change in outward direction. The localization shift in Experiment 2 was not significantly greater than zero in the absence, *M* = 0.58%, *SD* = 6.99%, *t*(11) = 0.29, *p* = 0.390, *d* = 0.17, or presence of reference objects, *M* = 4.37%, *SD* = 6.92%, *t*(11) = 2.19, *p* = 0.051, *d* = 1.32. In Experiment 3, the localization shift was not significant as well, neither when reference objects were absent (*M* = 1.02%, *SD* = 4.04%, *z* = −0.61, *p* = 0.545, *d* = −0.35) nor present, *M* = 3.92%, *SD* = 7.50%, *t*(11) = 1.81, *p* = 0.098, *d* = 1.09. Further, the localization change in Experiments 2 and 3 did not differ significantly, regardless of whether post-saccadic reference objects were absent, *t*(21.86) = 0.15, *p* = 0.879, *d* = 0.07, or present, *t*(17.61) = −0.19, *p* = 0.085, *d* = −0.09. These findings indicate that the direction of the color swap does not affect the perceived position of objects presented during the saccade preparation period. Further, the color swap did not lead to substantial changes in the perceived location of an object presented during the saccade preparation period. Figure 7 confirms the absence of a localization shift for trials during which the object array was removed from the screen. However, it also shows that, when the object array remains on screen, most participants exhibit a (non-significant) tendency to localize further outward at the end of the experiment, consistent with the pattern of results from Experiment 1.

#### Localization error

**Figure 8.**
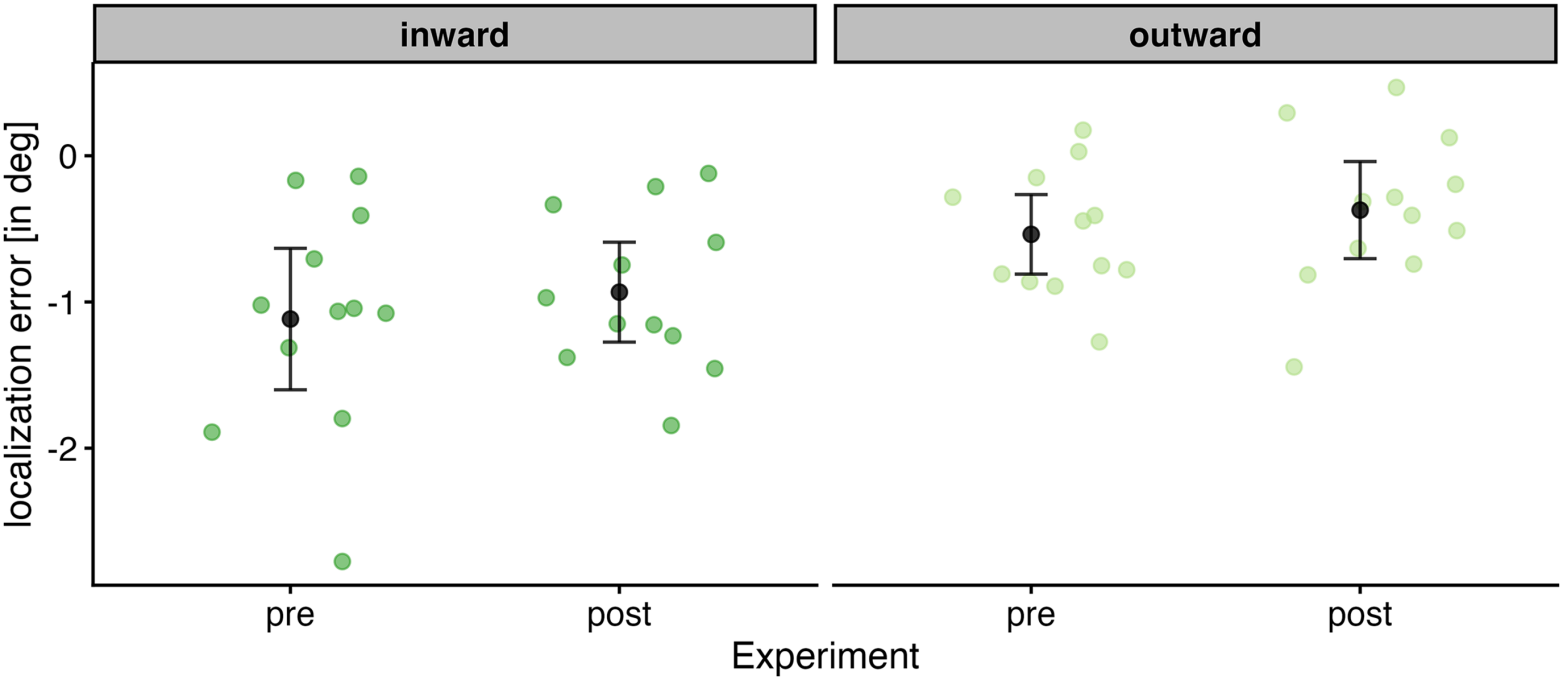
Localization error in Experiments 2 (inward color swap) and 3 (outward color swap). The black dots illustrate the average localization error in the respective experiment, depicted separately for localization judgments made before and after the adaptation phase. The bars depict the corresponding 95% confidence interval. The green dots illustrate the average localization error of each participant.

To test if a reduction of the localization error contributed to the localization change, we compared the localization error before and after the learning phase. The localization error in Experiment 2 did not change significantly from the beginning, *M* = 1.12°, *SD* = 0.76°, to the end (*M* = −0.93°, *SD* = 0.54°) of the experiment, *t*(11) = −1.68, *p* = 0.061, *d* = −0.51. The same is true for Experiment 3, beginning: *M* = −0.54, *SD* = 0.43; end: *M* = −0.37, *SD* = 0.52; *t*(11) = −1.39, *p* = 0.097, *d* = −0.42. However, the results confirmed the finding from Experiment 1 that the participants underestimated the eccentricity of the localization stimulus. This underestimation was significant at the beginning, Experiment 2: *t*(11) = −5.08, *p* < 0.001, *d* = −3.06; Experiment 3: *t*(11) = −4.35, *p* = 0.016, *d* = −2.62, and at the end, Experiment 2: *t*(11) = −6.01, *p* < 0.001, *d* = −3.62; Experiment 3: *t*(11) = −2.46, *p* = 0.016, *d* = −1.48. Although the reduction of this undershoot from before to after the learning phase is not significant in Experiments 2 and 3, the results are directionally consistent with the data from Experiment 1: the magnitude of the average undershoot tends to become smaller (Figure 8). The results further indicate that the underestimation of the eccentricity of the localization stimulus is more pronounced in Experiment 2 (*M* = −1.03,*SD* = 0.65) than in Experiment 3 (*M* = −0.45,*SD* = 0.48); *t*(42.06) = −3.47, *p* = 0.001, *d* = −1.07), potentially indicating that the intra-saccadic color swap in an inward direction might increase the tendency to underestimate the localization of the target eccentricity compared with a color swap in an outward direction. Taken together, the results suggest that the repeated intra-saccadic color swap does not affect the perceived location of objects. Otherwise, differences in localization change, dependent on the direction of the color swap, should emerge.

## General discussion

In the current study, the effect of different intra-saccadic manipulations on the oculomotor behavior and the perceived location of objects was investigated. The results confirm our previous finding that oculomotor learning does not depend on the intra-saccadic manipulation alone, but on a combination of the manipulation and the current task. More specifically, the results confirmed that task-relevant feature dimensions in the post-saccadic scene are prioritized for target selection and error evaluation (Heins & Lappe, 2022b). A different pattern of results emerged for the perceived object location: the change in perceived location that developed across the learning phase was dependent on the intra-saccadic manipulation. In the AS and ASwCS conditions that included an inward shift of the object array, the perceived location of objects shifted in inward direction. In the CS condition, however, a small but significant shift in perceived object location developed in an outward direction. In all conditions, the localization shift developed independently of the instruction and the corresponding oculomotor behavior.

Our first hypothesis stated that a subtle displacement of the object array would induce a localization shift in the direction of the array shift. The results from the AS condition confirmed this hypothesis: The participants localized the localization stimulus further inward after repeated intra-saccadic array shifts against saccade direction. The localization shift developed independently of the instruction. The information sources within the post-saccadic image, that is, the color of the target object, its shape, and its relative position to other objects within the array, were congruent and indicated unambiguously that the saccade had overshot the target object. The saccade gain decreased regardless of the instruction and, thus, the adaptive changes to the oculomotor behavior and object localization were congruent. The localization shift in direction of the array shift is consistent with previous research demonstrating changes in the perceived location of objects in response to continued and systematic intra-saccadic target displacements (e.g., Awater et al., 2005; Bahcall & Kowler, 1999; Bruno & Morrone, 2007; Collins et al., 2007; Schnier et al., 2010; Zimmermann & Lappe, 2009). The intra-saccadic array shift, similar to the displacement of an isolated target object, can be considered a sensory error. This sensory error does not lead to an abandonment of the assumption of a stable trans-saccadic world as the target step goes unnoticed, and, therefore, the pre- and post-saccadic target object are integrated into the percept of a stable object (Atsma et al., 2016). But what renders this kind of intra-saccadic manipulation so robust against detection? First, the change in absolute position of the object array is hard to detect because of saccadic suppression of displacement (Bridgeman et al., 1975; Li & Matin, 1990). Second, this manipulation leaves the spatial relation between the visible objects unchanged. There are no stationary reference objects or background cues against which the position of the object array before and after the saccade could be compared. The subtle intra-saccadic position change causes the entire visual world to appear at a retinal position that is inconsistent with the computed displacement of visual space during the saccade. Therefore, we term this kind of sensory error the global spatial error. According to the postdiction error model, its cause is attributed to erroneous motor performance or inaccurate spatial localization of the pre-saccadic target object. To reduce the error, three different synaptic gains adapt: visual gain, motor gain, and CD gain. Adaptation of the visual gain affects the transformation of the retinal input into the perceived pre-saccadic target location. Such changes to the pre-saccadic target localization have been reported in numerous studies (Collins et al., 2007; Schnier et al., 2010; Zimmermann & Lappe, 2010). Adaptation of the motor gain affects the transformation from the pre-saccadic target percept into the motor command, leading to the gradual shift in saccade landing position toward the displaced target position (Deubel et al., 1986; McLaughlin, 1967). In the case of inward target steps, the motor gain is assumed to decrease. The CD gain, which transforms the motor command into the computed displacement of space during the eye movements, increases. Due to the countervailing changes to motor gain and CD gain, the computed displacement of visual space underestimates the adaptive changes to the saccade vector, which leads to the adaptation-induced shift in direction of the target displacement (Masselink & Lappe, 2021). The data from the current study do not allow to us draw conclusions on the visual gain because we do not measure pre-saccadic localization. However, the data from the AS condition are consistent with the countervailing adaptation of motor gain and CD gain, as suggested by the postdiction error model.

Our second hypothesis stated that no change in perceived object location would develop following an obvious intra-saccadic color swap. Such an obvious color swap against saccade direction was applied in the CS condition. It produced a small but significant localization shift that, however, was against the direction of the color change. This change in perceived location developed regardless of the instruction, even though the saccade gain depended on the instruction. Following the color instruction, the saccade gain decreased, and following the shape instruction, a stable saccade gain was maintained. These results demonstrate that the participants used the ambiguous information regarding the target object within the post-saccadic image and flexibly selected the task-relevant information source to control their overt oculomotor behavior such that it complied with the instruction (Heins & Lappe, 2022b). This finding is in line with previous results indicating that changes to oculomotor behavior and perceived object location do not necessarily have to develop in parallel (Heins & Lappe, 2022a; Tyralla et al., 2023). But what about the localization shift in response to the intra-saccadic color swap? The color swap also induces a sensory error, because the red color appears at a retinal position that is inconsistent with the computed displacement of visual space during the saccade. However, this sensory error is different from the sensory error induced with the array shift in that it is easily detected. The color swap causes the red color to appear at another location in the post-saccadic than in the pre-saccadic visual scene, and the manipulation is obvious because it changes the position of a salient feature in spatial relation to reference objects. We therefore refer to this error as a local feature error. Because the manipulation is so easy to detect, the assumption of a stable trans-saccadic object might be quickly rejected (Atsma et al., 2016; Choi et al., 2025). Consequently, the local feature error might not be attributed internally, but to a change in the external world. Because both the assumption of a stable trans-saccadic world and internal error attribution are critical assumptions in the postdiction error model, no motor error should be computed or nullified. Congruently, we expected the absence of any systematic changes to object localization. The small outward shift in object localization does not align with this hypothesis. Perhaps this apparent shift in perceived location reflects an improvement in the localization task that is not related to the color swap itself. Initially, the eccentricity of the localization stimulus was underestimated for all three manipulations, leading to a negative localization error. Following the repeated displacement of the object array against saccade direction in the AS and ASwCS conditions, the tendency to underestimate the stimulus eccentricity increased, which caused the expected localization shift in inward direction. Following the repeated color swap in the CS condition, the tendency to underestimate the eccentricity of the localization stimulus decreased significantly. The overall localization error therefore increased from the beginning to the end of the experiment in the AS and ASwCS conditions, but decreased in the CS condition. Thus, the unexpected localization change in outward direction in the CS condition might reflect increased familiarity with the localization task and the intra-saccadic manipulation, that is, the obvious color swap, rather than the perceptual consequences of an oculomotor learning process. The results from Experiments 2 and 3 support this hypothesis because no significant localization change developed, regardless of the direction of the color swap. Instead, the data suggest that, as in Experiment 1, the participants initially underestimate the eccentricity of the localization stimulus. Although the subsequent reduction of the localization error reached significance only in Experiment 1, a trend to localize more accurately, and thus, further outward, is also evident in Experiments 2 and 3. What does that mean for the synaptic gains suggested by the postdiction error model? The local feature error poses an obvious violation of the assumption of a stable external world. As the postdicted motor error operates under this assumption, the local feature error might be disregarded for the computation of motor accuracy. Following the shape instruction, the saccade gain is thus easily kept stable. Following the color instruction, the shortened saccade vector might reflect the selection of another goal location in the scene that is better suited to obtain task success (Heins & Lappe, 2024). Motor learning from such task or reward errors supposedly involves identifying the motor command that maximizes the probability of success (Huang, Haith, Mazzoni, & Krakauer, 2011), but does not affect the localization of objects. The absence of opposing changes to motor gain and CD gain might provide an explanation for the lack of adaptation-induced changes to the post-saccadic localization.

Our third hypothesis stated that the combination of the array shift in saccade direction and the color swap against saccade direction would lead to a localization shift in direction of the array shift. The results confirm this hypothesis: Following the combination of an array shift against saccade direction and a color swap in saccade direction (ASwCS condition), a significant localization shift consistent with the direction of the AS developed. This localization shift was independent of the instruction. Following this combined manipulation, the information sources within the post-saccadic image were incongruent and therefore did not indicate the target object unambiguously. The oculomotor behavior following this intra-saccadic manipulation depended on the instruction, thus leading to incongruent changes to the oculomotor behavior and object localization. The participants adjusted their saccade such that they foveated the object with the target-defining feature. Following the shape instruction, participants decreased the saccade gain to look at the object with the correct shape. Following the color instruction, participants had to keep a stable gain to look at the red object. Although the gain was not entirely stable, it decreased less than following the position instruction, indicating compliance with the instruction. These results are in line with previous studies indicating that tasks modulate saccadic adaptation (Heins & Lappe, 2022a; Heins & Lappe, 2022b; Heins, Meermeier, & Lappe, 2019; Schütz, Kerzel, & Souto, 2014; Schütz & Souto, 2015; Souto & Schütz, 2020). In addition, we demonstrated in one of our previous works that the oculomotor behavior following intra-saccadic target displacements is dependent on the current task, while the localization shift is not. The localization shift develops in response to the subtle intra-saccadic displacement of the target object, regardless of the task or the corresponding oculomotor behavior (Heins & Lappe, 2022a). The current results from the ASwCS condition confirm that a localization shift develops in response to the subtle intra-saccadic displacement of the target object. It is consistent with the hypothesis that the displacement of the object array causes a global spatial error that inevitably leads to the postdiction of a motor error. This implicit process is independent of the overt motor behavior, and it disregards the color swap that violates the assumption of stable transsaccadic objects.

In both the CS and ASwCS conditions, the respective intra-saccadic manipulations lead to ambiguous post-saccadic information regarding the correct target object for the eye movement. Depending on the instruction, the participants either had to decrease the saccade gain or keep it stable to foveate the target and to comply with the task. The performance on the localization task was not affected by the overt oculomotor behavior, which is in line with the fourth hypothesis. But how can this divergence between the overt motor behavior and perceived object location be explained? We propose the following interplay of error signals as an explanation: the undetected intra-saccadic displacement of the object array in the ASwCS condition induces a global spatial error, indicating that the saccade was too large. Correspondingly, the motor and CD gain adapt to reduce the postdicted motor error, affecting the post-saccadic localization of objects. However, the overt oculomotor behavior does not necessarily reflect this adapted state of the oculomotor system. Following the color instruction, the automatic, adapted saccade might be suppressed in favor of a saccade that brings the eye close to the red object. In the CS condition, the obvious color swap induces the local feature error, which is attributed to a change in the external world. The overt oculomotor behavior is again largely independent of this sensory error and adjusts to the task demands. We therefore assume that the executed saccade nullifies a task error in both conditions. It is possible that those task-compliant saccades are guided by an artifical goal location, which might have been constructed postdictively as well. We demonstrated in a previous study that a pre-saccadic target representation can be built from post-saccadic information. It then served as a substitute for a physical target object and guided saccade targeting. Because this target representation was built from post-saccadic information only, the post-saccadic target location must have been postdicted into pre-saccadic space (Heins, Masselink, Scherer, & Lappe, 2023). Participants in our current study used the post-saccadic image to assess task success, that is, if they foveated the object with the correct feature value on the target-defining feature dimension. To improve their task performance in future trials, they might have used post-saccadic information about the position of the target object in relation to other objects to build an artificial goal location in the pre-saccadic space. Previous studies further indicated that, if the motor behavior required to reduce a task error was incongruent with the implicit adaptation following an internally attributed sensory error, the implicit adaptation developed latently. This latent adaptation becomes apparent in perceptual (Heins & Lappe, 2022a) or motor aftereffects of the learning process (Heins & Lappe, 2022b), and it might even interfere with task performance (Mazzoni & Krakauer, 2006). Our results therefore support the assumption that sensory errors finetune internal models (Shadmehr, Smith, & Krakauer, 2010; Wolpert, Ghahramani, & Jordan, 1995), whereas task errors indicate which behavior is best suited to improve the probability of task success (Huang et al., 2011). Postdictive processes might support the execution of saccades that maximize the probability of task success by generating an artificial goal location in pre-saccadic space.

## References

[bib1] Abel, L. A., Schmidt, D., Dell'Osso, L. F., & Daroff, R. B. (1978). Saccadic system plasticity in humans. *Annals of Neurology,* 4(4), 313–318, 10.1002/ana.410040405. 727736

[bib2] Atsma, J., Maij, F., Koppen, M., Irwin, D. E., & Medendorp, W. P. (2016). Causal inference for spatial constancy across saccades. *PLoS Computational Biology,* 12(3), e1004766, 10.1371/journal.pcbi.1004766. 26967730 PMC4788429

[bib3] Awater, H., Burr, D., Lappe, M., Morrone, M. C., & Goldberg, M. E. (2005). Effect of saccadic adaptation on localization of visual targets. *Journal of Neurophysiology,* 93(6), 3605–3614, 10.1152/jn.01013.2003. 15843478

[bib4] Bahcall, D. O., & Kowler, E. (1999). Illusory shifts in visual direction accompany adaptation of saccadic eye movements. *Nature,* 400(6747), 864–866, 10.1038/23693. 10476963

[bib5] Bahcall, D. O., & Kowler, E. (2000). The control of saccadic adaptation: Implications for the scanning of natural visual scenes. *Vision Research,* 40(20), 2779–2796, 10.1016/S0042-6989(00)00117-6. 10960651

[bib6] Becker, W. (1989). The neurobiology of saccadic eye movements. metrics. *Reviews of Oculomotor Research,* 3, 13–67. 2486323

[bib7] Brainard, D. H. (1997). The psychophysics toolbox. *Spatial Vision,* 10(4), 433–436, 10.1163/156856897X00357. 9176952

[bib8] Bridgeman, B., Hendry, D., & Stark, L. (1975). Failure to detect displacement of the visual world during saccadic eye movements. *Vision Research,* 15(6), 719–722, 10.1016/0042-6989(75)90290-4. 1138489

[bib9] Bruno, A., & Morrone, M. C. (2007). Influence of saccadic adaptation on spatial localization: Comparison of verbal and pointing reports. *Journal of Vision,* 7(5), 16, 10.1167/7.5.16. 18217856

[bib10] Cavanaugh, J., Berman, R. A., Joiner, W. M., & Wurtz, R. H. (2016). Saccadic corollary discharge underlies stable visual perception. *Journal of Neuroscience,* 36(1), 31–42, 10.1523/JNEUROSCI.2054-15.2016. 26740647 PMC4701964

[bib11] Choi, Y. M., Chiu, T.-Y., Ferreira, J., & Golomb, J. D. (2025). Maintaining visual stability in naturalistic scenes: The roles of trans-saccadic memory and default assumptions. *Cognition,* 262,106165, 10.1016/j.cognition.2025.106165. 40349562 PMC12225715

[bib12] Collins, T., Doré-Mazars, K., & Lappe, M. (2007). Motor space structures perceptual space: Evidence from human saccadic adaptation. *Brain Research,* 1172, 32–39, 10.1016/j.brainres.2007.07.040. 17803970

[bib13] Collins, T., Vergilino-Perez, D., Beauvillain, C., & Doré-Mazars, K. (2007). Saccadic adaptation depends on object selection: Evidence from between- and within-object saccadic eye movements. *Brain Research,* 1152, 95–105, 10.1016/j.brainres.2007.03.025. 17433271

[bib14] Collins, T., & Wallman, J. (2012). The relative importance of retinal error and prediction in saccadic adaptation. *Journal of Neurophysiology,* 107(12), 3342–3348, 10.1152/jn.00746.2011. 22442574 PMC3378410

[bib15] Crapse, T. B., & Sommer, M. A. (2008). The frontal eye field as a prediction map. In *Progress in brain research* (pp. 383–390, Vol. 171). New York: Elsevier, 10.1016/S0079-6123(08)00656-0. 18718330 PMC5152575

[bib16] Currie, C. B., McConkie, G. W., Carlson-Radvansky, L. A., & Irwin, D. E. (2000). The role of the saccade target object in the perception of a visually stable world. *Perception & Psychophysics,* 62(4), 673–683, 10.3758/BF03206914. 10883576

[bib17] Deubel, H., Wolf, W., & Hauske, G. (1986). Adaptive gain control of saccadic eye movements. *Human Neurobiology,* 5(4), 245–253. 3818374

[bib18] Frossard, J., & Renaud, O. (2021). Permutation tests for regression, ANOVA, and comparison of signals: The permuco package. *Journal of Statistical Software,* 99(15), 10.18637/jss.v099.i15.

[bib19] Georg, K., & Lappe, M. (2009). Effects of saccadic adaptation on visual localization before and during saccades. *Experimental Brain Research,* 192(1), 9–23, 10.1007/s00221-008-1546-y. 18716763

[bib20] Heins, F., & Lappe, M. (2022a). Mislocalization after inhibition of saccadic adaptation. *Journal of Vision,* 22(8), 3, 10.1167/jov.22.8.3. PMC9290319 35834378

[bib21] Heins, F., & Lappe, M. (2022b). Flexible use of post-saccadic visual feedback in oculomotor learning. *Journal of Vision,* 22(1), 3, 10.1167/jov.22.1.3. PMC8742532 34994785

[bib22] Heins, F., & Lappe, M. (2024). Oculomotor behavior can be adjusted on the basis of artificial feedback signals indicating externally caused errors. *PLoS One,* 19(5), e0302872, 10.1371/journal.pone.0302872. 38768134 PMC11104623

[bib23] Heins, F., Masselink, J., Scherer, J.-N., & Lappe, M. (2023). Adaptive changes to saccade amplitude and target localization do not require pre-saccadic target visibility. *Scientific Reports,* 13(1), 8315, 10.1038/s41598-023-35434-8. 37221275 PMC10205735

[bib24] Heins, F., Meermeier, A., & Lappe, M. (2019). Volitional control of saccadic adaptation. *PLoS One,* 14(1), e0210020, 10.1371/journal.pone.0210020. 30629610 PMC6328110

[bib25] Henson, D. B. (1979). Investigation into corrective saccadic eye movements for refixation amplitudes of 10 degrees and below. *Vision Research,* 19(1), 57–61, 10.1016/0042-6989(79)90121-4. 419701

[bib26] Huang, V. S., Haith, A., Mazzoni, P., & Krakauer, J. W. (2011). Rethinking motor learning and savings in adaptation paradigms: Model-free memory for successful actions combines with internal models. *Neuron,* 70(4), 787–801, 10.1016/j.neuron.2011.04.012. 21609832 PMC3134523

[bib27] Kherad-Pajouh, S., & Renaud, O. (2015). A general permutation approach for analyzing repeated measures ANOVA and mixed-model designs. *Statistical Papers,* 56(4), 947–967, 10.1007/s00362-014-0617-3.

[bib28] Kleiner, M., Brainard, D., Pelli, D., Ingling, A., Murray, R., & Broussard, C. (2007). What's new in psychtoolbox-3? *Perception,* 36(14).

[bib29] Kommerell, G., Olivier, D., & Theopold, H. (1976). Adaptive programming of phasic and tonic components in saccadic eye movements. investigations of patients with abducens palsy. *Investigative Ophthalmology,* 15(8), 657–660. 955831

[bib30] Lappe, M., Awater, H., & Krekelberg, B. (2000). Postsaccadic visual references generate presaccadic compression of space. *Nature,* 403(6772), 892–895, 10.1038/35002588. 10706286

[bib31] Lenth, R. V. (2025). Emmeans: Estimated marginal means, aka least-squares means [R package version 1.11.1-00001], https://github.com/rvlenth/emmeans.

[bib32] Li,W., & Matin, L. (1990). The influence of saccade length on the saccadic suppression of displacement detection. *Perception & Psychophysics,* 48(5), 453–458, 10.3758/BF03211589. 2247328

[bib33] Madelain, L., Harwood,M. R., Herman, J. P., & Wallman, J. (2010). Saccade adaptation is unhampered by distractors. *Journal of Vision,* 10(12), 29, 10.1167/10.12.29. 21047761

[bib34] Masselink, J., & Lappe, M. (2021). Visuomotor learning from postdictive motor error. *eLife,* 10, e64278, 10.7554/eLife.64278. 33687328 PMC8057815

[bib35] Mazzoni, P., & Krakauer, J. (2006). An implicit plan overrides an explicit strategy during visuomotor adaptation. *Journal of Neuroscience,* 26(14), 3642–3645, 10.1523/JNEUROSCI.5317-05.2006. 16597717 PMC6674132

[bib36] McConkie, G. W., & Currie, C. B. (1996). Visual stability across saccades while viewing complex pictures. *Journal of Experimental Psychology: Human Perception and Performance,* 22(3), 563, 10.1037/0096-1523.22.3.563. 8666953

[bib37] McLaughlin, S. C. (1967). Parametric adjustment in saccadic eye movements. *Perception & Psychophysics,* 2(8), 359–362, 10.3758/BF03210071.

[bib38] Melcher, D., & Colby, C. L. (2008). Trans-saccadic perception. *Trends in Cognitive Sciences,* 12(12), 466–473, 10.1016/j.tics.2008.09.003. 18951831

[bib39] Miller, J. M., Anstis, T., & Templeton, W. B. (1981). Saccadic plasticity: Parametric adaptive control by retinal feedback. *Journal of Experimental Psychology: Human Perception and Performance,* 7(2), 356–366, 10.1037/0096-1523.7.2.356. 6453929

[bib40] Niemeier, M., Crawford, J. D., & Tweed, D. B. (2003). Optimal transsaccadic integration explains distorted spatial perception. *Nature,* 422(6927), 76–80, 10.1038/nature01439. 12621435

[bib41] Noto, C. T., & Robinson, F. R. (2001). Visual error is the stimulus for saccade gain adaptation. *Cognitive Brain Research,* 12(2), 301–305, 10.1016/S0926-6410(01)00062-3. 11587898

[bib42] Richard, A. M., Luck, S. J., & Hollingworth, A. (2008). Establishing object correspondence across eye movements: Flexible use of spatiotemporal and surface feature information. *Cognition,* 109(1), 66–88, 10.1016/j.cognition.2008.07.004. 18760406 PMC2650243

[bib43] Ross, J., Morrone, M. C., & Burr, D. C. (1997). Compression of visual space before saccades. *Nature,* 386(6625), 598–601, 10.1038/386598a0. 9121581

[bib44] Schnier, F., Zimmermann, E., & Lappe, M. (2010). Adaptation and mislocalization fields for saccadic outward adaptation in humans. *Journal of Eye Movement Research,* 3(3), 10.16910/jemr.3.3.4.

[bib45] Schütz, A. C., Kerzel, D., & Souto, D. (2014). Saccadic adaptation induced by a perceptual task. *Journal of Vision,* 14(5), 4, 10.1167/14.5.4. 24799623

[bib46] Schütz, A. C., & Souto, D. (2015). Perceptual task induces saccadic adaptation by target selection. *Frontiers in Human Neuroscience,* 9, 10.3389/fnhum.2015.00566. PMC4611985 26539095

[bib47] Scudder, C. A., Batourina, E. Y., & Tunder, G. S. (1998). Comparison of two methods of producing adaptation of saccade size and implications for the site of plasticity. *Journal of Neurophysiology,* 79(2), 704–715, 10.1152/jn.1998.79.2.704. 9463434

[bib48] Semmlow, J. L., Gauthier, G. M., & Vercher, J.-L. (1989). Mechanisms of short-term saccadic adaptation. *Journal of Experimental Psychology: Human Perception and Performance,* 15(2), 249–258, 10.1037/0096-1523.15.2.249. 2525598

[bib49] Shadmehr, R., Smith, M. A., & Krakauer, J. W. (2010). Error correction, sensory prediction, and adaptation in motor control. *Annual Review of Neuroscience,* 33(1), 89–108, 10.1146/annurev-neuro-060909-153135. 20367317

[bib50] Souto, D., & Schütz, A. C. (2020). Task-relevance is causal in eye movement learning and adaptation. In *Psychology of learning and motivation* (pp. 157–193, Vol. 73). New York: Elsevier, 10.1016/bs.plm.2020.06.002.

[bib51] Straube, A., Fuchs, A. F., Usher, S., & Robinson, F. R. (1997). Characteristics of saccadic gain adaptation in rhesus macaques. *Journal of Neurophysiology,* 77(2), 874–895, 10.1152/jn.1997.77.2.874. 9065856

[bib52] Tas, A. C., Moore, C. M., & Hollingworth, A. (2012). An object-mediated updating account of insensitivity to transsaccadic change. *Journal of Vision,* 12(11), 18, 10.1167/12.11.18. PMC3720035 23092946

[bib53] Tyralla, S., Pomè, A., & Zimmermann, E. (2023). Motor recalibration of visual and saccadic maps. *Proceedings of the Royal Society B,* 290(1994), 10.1098/rspb.2022.2566. PMC9975659 36855869

[bib54] Van Der Stigchel, S., & Hollingworth, A. (2018). Visuospatial working memory as a fundamental component of the eye movement system. *Current Directions in Psychological Science,* 27(2), 136–143, 10.1177/0963721417741710. 29805202 PMC5967619

[bib55] Wallman, J., & Fuchs, A. F. (1998). Saccadic gain modification: Visual error drives motor adaptation. *Journal of Neurophysiology,* 80(5), 2405–2416, 10.1152/jn.1998.80.5.2405. 9819252

[bib56] Wolpert, D. M., Ghahramani, Z., & Jordan, M. I. (1995). An internal model for sensorimotor integration. *Science* (New York, NY), 269(5232), 1880–1882, 10.1126/science.7569931. 7569931

[bib57] Wong, A. L., & Shelhamer, M. (2011). Sensorimotor adaptation error signals are derived from realistic predictions of movement outcomes. *Journal of Neurophysiology,* 105(3), 1130–1140, 10.1152/jn.00394.2010. 21123665 PMC3074418

[bib58] Wurtz, R. H. (2018). Corollary discharge contributions to perceptual continuity across saccades. *Annual Review of Vision Science,* 4, 215–237, 10.1146/annurev-vision-102016-061207. 30222532

[bib59] Zimmermann, E., & Lappe, M. (2009). Mislocalization of flashed and stationary visual stimuli after adaptation of reactive and scanning saccades. Journal of Neuroscience, 29(35), 11055–11064, 10.1523/JNEUROSCI.1604-09.2009. 19726664 PMC6665523

[bib60] Zimmermann, E., & Lappe, M. (2010). Motor signals in visual localization. *Journal of Vision,* 10(6), 2, 10.1167/10.6.2. 20884551

